# Treatment of severe cow’s milk allergy with omalizumab in an adult 

**DOI:** 10.5414/ALX02372E

**Published:** 2023-02-03

**Authors:** Benjamin Klein, Jan Christoph Simon, Regina Treudler

**Affiliations:** 1 Department of Dermatology, Venereology, and Allergology, and LICA-CAC, University Hospital Leipzig, Leipzig, Leipzig, Germany

**Keywords:** food allergy, omalizumab, cow’s milk allergy

## Abstract

Background: The therapy of severe food allergy so far consists mainly of allergen abstinence and emergency treatment. The use of anti-IgE antibodies represents a promising therapy. Case report: We report on a 22-year-old male with severe cow’s milk allergy with multiple anaphylactic reactions, known since infancy and persisting into adulthood with sometimes severe immediate-type reactions on accidental ingestion. The prick test for native whole milk was positive, the CAP-FEIA was also positive for milk protein, mare’s milk, whey, sheep’s milk whey as well as Bos d4, Bos d5, and Bos d8 and blue cheese; total IgE was 1,265 kU/L. The patient’s history included well-controlled bronchial asthma. An off-label therapy with omalizumab (3 × 150 mg/month SC) and cetirizine 10 mg once daily was initiated. Under this therapy, we performed a double-blind oral exposure test to cow’s milk in the patient after long term. Thereby 14 mL could be tolerated. After consumption of 30 mL of cow’s milk, urticaria, dyspnea, and angioedema occurred. Conclusion: Under therapy with omalizumab, an increase of the tolerance to cow’s milk was shown in our patient. As a consequence, reactions during accidental consumption could be prevented.

## Introduction 

The treatment of severe food allergy is a therapeutic challenge. Avoidance is not completely possible in all cases, and when even accidental consumption leads to anaphylactic reactions, new strategies are sought in addition to the use of emergency medication [12]. 

In Europe, cow’s milk allergy represents a common IgE-mediated food allergy in childhood with an incidence of 0.54% [8]. Tolerance is reported to develop in ~ 70% of cases after 1 year [8]. After 15 years, 79 – 97% of affected individuals show tolerance development in observational studies [3, 9]. Risk factors favoring persistence of allergy represent elevated allergen-specific IgE levels, IgE levels against heat-stable proteins (see below), other IgE-mediated food allergies, coexisting bronchial asthma, and allergic rhinitis [9]. 

Various cow’s milk components have so far been identified as triggers of an IgE-mediated reaction, distinguishing heat-stable and labile proteins. Milk protein consists of 80% casein and 20% whey protein. Heat-stable proteins are the casein portion (caseins, Bos d8 – 12), which is more than 90% cross-reactive to other animal milk (sheep, goat, and camel milk) [5]. Labile proteins include the whey proteins α-lactalbumin (Bos d4), β-lactoglobulin (Bos d5), bovine serum albumin (Bos d6), and lactoferrin (Bos d lactoferrin). In this context, bovine serum albumin (Bos d6) is cross-reactive with beef [5]. An overview of the different allergens of milk and their properties is given in Table 1. 

The prevention of cow’s milk allergy was recently investigated in a study in which the early (1^st^ – 2^nd^ month of life) introduction of a cow’s milk-based formulation significantly decreased the development of cow’s milk allergy [7]. While nutritional alternatives (formula feeding/hydrolysate formulas and calcium supplementation) are necessary in infancy and early childhood, therapy for cow’s milk allergy in adulthood is mainly realized by allergen avoidance and the presence of an emergency kit [5]. Here, we report the case of an adult with severe cow’s milk allergy under treatment with omalizumab. 

## Case report 

A 22-year-old patient reported generalized urticaria and dyspnea after consumption of cow’s milk in the 1^st^ year of life. Prior to that, he had been continuously breastfed for 6.5 months. Since infancy, there was a period of abstinence with prescription of an emergency kit. Sensitization to several milk protein proteins (Bos d 4, Bos d 5, Bos d 6, and Bos d 8) was monitored periodically and was persistent. At the age of 22 years, the patient had unintentionally eaten a steak from the grill, on which cheese had been placed beforehand, resulting in a reaction of the patient with shortness of breath, a feeling of obstruction in his throat as well as generalized urticaria and, later, unconsciousness. 

In addition, the patient’s history included atopic eczema in childhood and he had developed allergic bronchial asthma with type I sensitization to mugwort and cat dander since the age of 13, which was stable under combination therapy with salmeterol and fluticasone 1-0-0 per inhalation. 

The prick test was positive to whole milk and cow’s milk (ALK) (Table 2). The total IgE was 1,265 kU/L, and an IgE sensitization was present to milk protein, mare’s milk, whey, sheep’s milk whey as well as Bos d4, Bos d5, and Bos d8, and blue cheese (Table 2). 

After reimbursement had been approved by the health insurance, an off-label therapy with omalizumab 3 × 150 mg SC every 4 weeks was started with absence of adverse drug reactions. However, in the following year, the patient reacted with a severe asthma attack as well as angioedema 20 minutes after eating Asian food, whereupon the emergency kit had to be applied. He noted that there may have been whole milk in this meal instead of the soy milk he had wanted, but was now highly distressed by this strong reaction. 

## Results 

**Figure 1.**
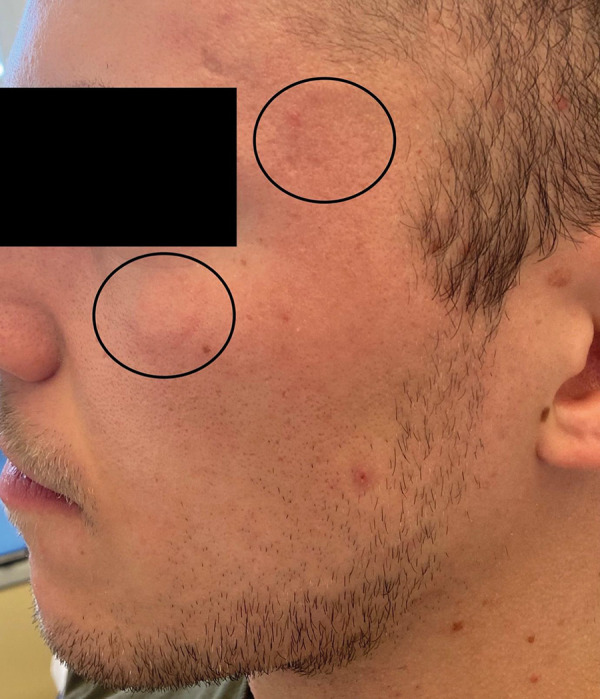
Occurrence of wheals (circles) after consumption of 30 mL cow’s milk under omalizumab in DBPCFC.

We performed a double-blind placebo-controlled food challenge (DBPCFC) with native whole milk during an inpatient stay under therapy with omalizumab (last applied 2 weeks before) and regular intake of cetirizine 10 mg 1-0-0. Doses included 0.1, 0.3, 1, 3, 10, and 30 mL of whole milk. Upon consumption of the last dose of 30ml of native whole milk, our patient developed facial wheals (Figure 1), low-grade angioedema, and subjective shortness of breath without a significant decrease in peak flow. Therefore, administration of 100 mg prednisolone and 4 mg dimethindene intravenously was necessary, under which the symptoms regressed. In summary, a cumulative dose of 14.4 mL of native whole milk was tolerated before the reaction. In the further follow-up of 2 years, no anaphylactic reactions occurred again under omalizumab. 

## Discussion 

This case illustrates how an increase of the tolerance to cow’s milk is achieved by therapy with cetirizine and omalizumab. While traces had previously led to severe immediate-type reactions in our patient, 14.4 mL are now tolerated without symptoms. Retrospectively, we assume a substitution of soy milk by cow’s milk (> 15 mL) for the anaphylactic reaction that occurred after the Asian meal. Oral immunotherapy (OIT) with cow’s milk under omalizumab was not desired by the patient, as he did not express a need for dairy products. This will be re-evaluated during every follow-up consultation in our allergy department. 

Since IgE-mediated cow’s milk allergy in childhood has a favorable prognosis with regard to tolerance development, annual monitoring of tolerance after 1 year of age is recommended. Initially, this can be done gradually by introducing highly heated dairy products (bakery products, chocolate), which are tolerated by ~ 75% of patients with past anaphylactic reactions [5]. Our patient represents one of the few cases with persistence of cow’s milk allergy into adulthood. He exhibited typical risk factors such as high total IgE, high specific IgE levels against heat-stable proteins of cow’s milk, and a history of bronchial asthma. Cow’s milk allergy in adulthood leads to severe anaphylactic reactions in up to 25% of cases as seen in our patient. 

Overall, the treatment of severe IgE-mediated food allergies with anti-IgE antibodies represents a promising therapeutic approach. An increase in tolerance with this therapy was reported in peanut allergy [4]. Furthermore, omalizumab facilitated the tolerability of OIT in IgE-mediated food allergy [1, 2, 6]. Our case demonstrates the effective therapy of cow’s milk allergy with omalizumab in an adult and reinforces previous results. 

With regard to allergen-specific immunotherapy in cow’s milk allergy, there are no commercial preparations available to date. The efficacy and safety of OIT with omalizumab was investigated in an American randomized, placebo-controlled, double-blind (RPCDB) study in 2016 with 75 patients [11]. In this study, treatment with omalizumab showed significantly fewer adverse events and symptom-related emergency management compared to placebo [11]. Regarding efficacy, assessed by occurring desensitization and sustained unresponsiveness, there were no significant differences comparing patients on omalizumab versus placebo after oral food provocation [11]. This may reflect the spontaneous development of tolerance in a large proportion of affected individuals. It should be noted that the study by Wood et al. [11] excluded patients with severe anaphylactic reactions to cow’s milk, possibly underestimating the outcomes of patients who would benefit from omalizumab. 

Another RPCDB study from 2017 investigated the response of OIT with microwaved cow’s milk 8 weeks after therapy with omalizumab versus complete avoidance (placebo) in 16 patients with high specific IgE levels to cow’s milk proteins [10]. Here, 5 of the 10 patients treated with omalizumab were affected by severe anaphylaxis [10]. There were significant results regarding desensitization (prick test, specific IgE) and clinical symptoms in omalizumab-treated patients vs. placebo [10]. However, it should be noted that the placebo group had to perform complete avoidance first and subsequently, cow’s milk exposure was performed. Hence, comparability with respect to OIT can only be assessed to a limited extent [10]. 

Taken together, previous study results show increased safety of OIT with omalizumab in cow’s milk allergy. However, data from adult patients with cow’s milk allergy and OIT under omalizumab have not been published yet. In our case, OIT was not performed because the patient did not express a need for dairy products and omalizumab prevented an anaphylactic reaction to unintentional consumption of cow’s milk. Currently, a multicenter, RPCDB phase 3 trial is currently evaluating the efficacy and safety of the anti-IgE antibody ligelizumab (240 mg and 120 mg SC every 4 weeks) in patients with IgE-mediated peanut allergy (NCT04984876; clinicaltrials.gov). This specific anti-IgE antibody could possibly be used in other food allergies. Our case highlights omalizumab as an option to be discussed in individual cases of food allergy, especially in patients with severe symptoms and life-threatening anaphylactic reactions. 

## Funding 

None. 

## Conflict of interest 

Benjamin Klein: no conflicts of interest, Regina Treudler: COI: AbbVie, ALK-Abello, Leopharma, Novartis, Pfizer, Sanofi, Takeda, not in relation to this work. 

**Table 1.** Overview of important allergens of cow’s milk [5].

Milk protein	Recombinant allergen	Heat stability	Cross-reactivity
Whey	α-lactalbumin, Bos d 4	No	Unknown
	β-lactoglobulin, Bos d 5	No	Unknown
	Bovine serum albumine, Bos d 6	No	15 – 20% with beef
	Lactoferrin, Bos d lactoferrin	No	
Casein	Casein, Bos d 8	Yes	> 90% with other animal milk
	α S1-casein, Bos d 9	Yes	
	α S2-casein, Bos d 10	Yes	
	β-casein, Bos d 11	Yes	
	κ-casein	Yes	

**Table 2.** Sensitization to cow’s milk and milk proteins.

**Prick test:**	**Wheal/flare**	
Cow’s milk (ALK) Native whole cow milk Beef	10 mm/> 20 mm > 20 mm/> 20 mm 0 mm/0 mm	
**Molecular diagnostics:**	**Specific IgE (kU/L)**	**CAP class**
Total IgE 1,265 kU/L Specific IgE diagnostics: Milk protein: Whey: Bos d 4 (α-lactoglobulin) Bos d 5 (β-lactalbumin) Bos d 8 (casein) Sheep milk Goat milk Mold cheese Mare’s milk Beef d 1, Bet v 1, Alpha1,3-Gal	> 100.00 > 100.00 > 100.00 > 100.00 > 100.00 > 100.00 > 100.00 > 100.00 > 100.00 2.61 0.19	CAP 6 CAP 6 CAP 6 CAP 6 CAP 6 CAP 6 CAP 6 CAP 6 CAP 6 CAP 2 Each CAP 0
